# Clinical and Pathological Notes from a Breeding Station

**Published:** 1889-07

**Authors:** Wesley Mills

**Affiliations:** Professor of Physiology in McGill University and the Veterinary College, Montreal, Canada


### ART. XVI.—CLINICAL AND PATHOLOGICAL NOTES FROM A BREEDING STATION.

### I. SCROFULA AND TUBERCULOSIS IN BIRDS.

BY WESLEY MILLS, M.A., M.D.,
*Professor of Physiology in McGill University and the Veterinary College, Montreal, Canada.*

AT my place of residence in a suburb of this city, I keep a large number of animals, especially thorough-bred fowls and pigeons, with the object primarily of studying the subject of breeding, heredity, comparative psychology, etc. Naturally among so many, especially "fancy" or pure bred birds, cases of disease will occur, however well they may be cared for. Nor do I altogether regret it inasmuch as it throws not a little light on the essential nature of disease in general, that is to the relations between environment and the abnormal conditions of the organism.

So far as I am aware very little has been written on this continent on the diseases of birds. It has occurred to me, therefore, that even meagre notes from time to time may not be unacceptable. In this communication I propose to confine myself chiefly to scrofula and tuberculosis in pigeons as they have fallen under my own observation. The following cases will afford a basis for a consideration of the subject.

1. *Oriental Roller hen*, age not known, probably three to four years; color black. Had laid in the summer of 1888, during which time she was in my possession laid many eggs and hatched out several young ones, three of which are now alive and breeding. She seemed well till shortly before death, *i. e.*, to say till eight or ten days before the fatal issue. Was treated with some special food (hempseed) and Prarish's hypophosphites. For some days prior to death ate but little. Died on October 26th.

*Autopsy* showed complete careous degeneration of the ovary and oviduct. It will be remembered there is but one functionally active in birds.

2. *Fantail hen*, color yellow; age unknown; a delicate bird. Not breedding with its mate the birds were given a couple of eggs from another nest, which they duly hatched, and fed the young well. This was done with the object of getting the birds to breed, and is a plan sometimes successful; apparently it did stimulate the hen. She however never laid, though to appearances "broody." Bird seemed dull for two or three days, eating but little. Placed her in a large cage apart, and administered cod-liver oil. She died on September 24th.

*Autopsy* revealed cheesy mesenteric glands, and in the oviduct were what seemed like two old yolks of eggs. Bird had lost a good deal of flesh.

3. *Barb cock*, color black; age four to five years probably, if not more, would be called "old." This bird had been dull for ten to twelve days; died about December 28th. Had not had much treatment.

*Autopsy*, tubercle in various organs. Left testicle a caseous mass.

4. *Fantail cock*, color black; "old"—exact age unknown.

This bird was visibly ailing for a week at least; had marked diarrhœa. Given a little tonic treatment. Became somewhat thin. Died on November —.

*Autopsy* showed that the most pronounced lesion was caseous degeneration of the testicles.

5. *Jacobin cock*, color white; a young bird, the last bred of the season; hatched August 14th, and by September 16th was out of the nest and taking care of itself in great part. It was interesting as being bred from two reds, illustrating the principle of *reversion*. Though dull for a little while I did not suspect the case to be a serious one, and did not give it much treatment. Death about November 2d.

*Autopsy* gave evidence of the most extensive tuberculous lesions in all stages; douptful if any organ escaped. There were abundance of coagulated exudation and numerous adhesions. The liver seemed to be enlarged and the apex of the heart to be squeezed between its main lobes. The bird was fairly well nourished.

From such experience as I have thus far had tuberculosis seems to be much more common among pure bred pigeons than in the corresponding thoroughbred fowls.

In order to understand this, one must bear in mind that the breeding of pigeons is of a very exhaustive nature. If kept warm and well-fed they will breed during almost every month in the year, and some persons allow them to keep at it as they will. Then pigeons not only feed the young by disgorging into their crops the food from their own, but supply a special secretion from the crop; so that taking into account the number of eggs laid, the large amount of time spent on the nest, and the feeding process, it is not difficult to understand why breeding is so exhaustive.

I know that some of the birds reported above were allowed to breed as they would, summer and winter, before they came into my hands.

Again, in both fowls and pigeons, indeed in all kinds of birds especially when confined, the moulting season is most trying. There is nothing comparable to it in severity among mammals. When we consider that every feather is renewed within a few weeks it is apparent that an extraordinary amount of blood and nervous energy are directed to the skin with a corresponding diversion from other and vital organs. I have actually seen bleeding from the roots of the feathers, and to such an extent in one case that I suspected the bird (a young cockerel) had been injured.

My pigeons were in active moult last year from early in August till October, during which time none of them should have been allowed to breed. Late breeding, and previous over-breeding combined with the exhaustion of the moult, explain a great deal in most of the cases referred to above. It will be noticed that the deaths all occurred after the moulting season had set in.

The birds that suffered did not happen to belong to the most delicate varieties. My flocks then contained few of the latter class. But during our long and severe winter in Montreal, the older birds show much less resistance than the young ones. I mean by "older," birds past their prime. In other words age and climate are important factors in the causation of disease. The nutritive processes generally fail under these circumstances.

One of the facts that is strikingly exemplified in these cases is that the over-taxed generative system suffers primarily, and apparently in some cases exclusively so far as the tubercle or scrofula is concerned. I am not in a position to say whether in all of the above cases there was actual presence of tubercle bacilli or whether in some of them there was merely a rapid fatty degeneration from wearing out of the protoplasm of the cells. So quickly do birds succumb that it is not possible to be aware, it would seem, of the exact period of the onset of the disease; that is to say, the lesion either runs a course of extreme rapidity, which it certainly does in some cases, or it may be present and the bird manifest no well-marked signs of disease for some time.

In even the fifth case, with the most extensive tuberculosis, the bird seemed quite well till within at most three weeks of its death, and ate its food up to the last two or three days, and some even then. Now in this case I feel satisfied that the whole course of the disease did not extend over more than three weeks. It may be worth while to state that though all the offspring of the pair from which this bird was bred were quite healthy, one of the parents showed signs of failure in some directions before producing the egg from which the last one was hatched. Besides, the disease occurred during its first moult, a notably trying time. I hope to publish some notes on the heredity of disease later. It may be mentioned here that in one case the death of an adult bird (overbred) occurred during the moulting season apparently solely from anæmia, the explanation of which is to be sought in the drain of this period.

Case 5, shows clearly that a bird may be the subject of the most extensive tuberculosis and yet remain fairly well nourished up to the date of its death. I have reported only fatal cases ; it is not to be inferred that treatment is useless, however. This may be referred to again.

The most obvious and important inferences that seem to be deducible from the above cases are :—

1. Tuberculosis (and scrofula) run in birds a very rapid course.

2. Symptoms of a serious nature may be absent till within a few days of death.

3. Even death may result without pronounced emaciation.

4. The above cases illustrate the general law that in disease including tuberculosis the condition of the organism practically determines whether it shall arise or not.

5. The moult is a most serious drain on the organism of the bird, hence the frequency of disease during and soon after this event.

6. In pigeons, probably in hen birds, the generative organs are especially liable to a form of disease which ends in fatty degeneration.

7. Over-breeding is an important predisposing cause of disease, particularly of tuberculosis.

